# Scalable, Low‐Cost, Freeze‐Resistant Hydrogels by Alkali‐Polyphenol‐Triggered Polymerization for Low‐Temperature Self‐Powered Triboelectric Sensor

**DOI:** 10.1002/advs.74924

**Published:** 2026-03-23

**Authors:** Mengting Zhou, Yaru Bao, Chunpeng Li, Caicai Li, Gaobo Lou, Shaochao Sun, Zhezhe Zhou, Gaojie Jiao, Xiaochun Zhang, Pingan Song, Dan Sun

**Affiliations:** ^1^ College of Chemistry and Materials Engineering Zhejiang A&F University Hangzhou China; ^2^ School of Optoelectronic Materials & Technology Key Laboratory of Flexible Optoelectronic Materials and Technology (Ministry of Education) Jianghan University Wuhan China; ^3^ Guangxi Key Laboratory of Clean Pulp & Papermaking and Pollution Control School of Light Industry and Food Engineering Guangxi University Nanning China; ^4^ Centre For Future Materials School of Science Engineering and Digital Science University of Southern Queensland Springfield Australia; ^5^ State Key Laboratory of Advanced Papermaking and Paper‐based Materials South China University of Technology Guangzhou China

**Keywords:** alkali‐polyphenol, freeze‐resistant hydrogel, low‐temperature operation, multifunctional initiation, rapid polymerization, triboelectric nanogenerator

## Abstract

Hydrogels are promising flexible and conductive electrodes for triboelectric nanogenerators (TENGs), but their practical application is limited by cumbersome, high‐cost fabrication processes and inferior frost resistance. Herein, a novel multifunctional alkali‐polyphenol (DESL‐OH^−^) self‐catalytic system is artfully designed to drive the rapid ambient polymerization of α‐methacrylic acid (MAA) and hydroxyethyl acrylate (HEA) monomers, yielding a scalable, low‐cost hydrogel with exceptional cryo‐tolerance (−40°C), robust interfacial adhesion (0.065 MPa on paper), high conductivity (2.8 mS cm^−1^), and optical transparency (>88%) within two min. Its anti‐freezing performance stems from alkali‐driven HEA hydrolysis to generate ethylene glycol (EG), forming an endogenous EG‐water cosolvent system that disrupts the ordered tetrahedral arrangement of water molecules via hydrogen bonding interactions, suppresses ice nucleation, and thus maintains hydrogel functionality at extreme low temperatures. Harnessing these superior attributes, the hydrogel is assembled into a triboelectric nanogenerator (H‐TENG) that operates stably at −40°C (open‐circuit voltage = 154 V, short‐circuit current = 1.00 µA, transferred charge = 34.6 nC), serving as both a reliable power supply for commercial electronics and a high‐sensitivity tactile sensor for real‐time human motion monitoring. This work establishes an efficient, sustainable fabrication paradigm for designing anti‐freezing hydrogels devoid of exogenous cryoprotectants and provides critical insights for the development of next‐generation wearable energy harvesters.

## Graphical abstract

Leveraging the multidimensional self‐catalytic merits of the alkali‐polyphenol synergistic self‐catalytic platform, this work delineates the intrinsic mechanism governing the regulation of hydrogel properties and puts forward an innovative design paradigm for frost‐tolerant electrode materials, providing critical scientific insights and technical underpinnings to propel the practical applications of TENGs in extremely cold environments.

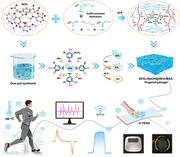

## 1 Introduction

The burgeoning advancement of flexible and wearable electronics has driven growing demand for sustainable and efficient energy supplies [1, 2, 3]. Conventional energy systems, however, suffer from limitations such as poor frost resistance [4], environmental concerns [5], and low energy conversion efficiency [6], which prevent them from meeting the critical requirements of wearable electronic devices. In response, researchers have stepped up efforts to develop flexible power sources and eco‐friendly energy systems tailored to wearable devices, aiming to improve their performance, stability, and application scope to address the escalating demand for sustainable energy [7]. Among these technologies, triboelectric nanogenerators (TENGs) can harvest low‐frequency mechanical energy through triboelectric and electrostatic induction effects and effectively convert it into electrical energy [6]. They also feature high output power density, light weight, low cost, and ease of fabrication, collectively providing transformative solutions for self‐powered wearable devices [8, 9]. Therefore, TENGs are regarded as one of the most promising energy technologies in the new era, capable of functioning as both energy supply units and self‐powered sensor units [10].

In addition to triboelectric materials, electrodes are also vital for the flexible and wearable applications of TENGs [11]. Specifically, the construction of stretchable TENGs relies heavily on flexible and stretchable electrodes, which enable their efficient integration into portable electronic devices. Traditional TENGs typically employ metallic conductors such as gold (Au), silver (Ag), aluminum (Al), and copper (Cu) as electrode layers [12, 13]. Despite their high conductivity of these metal electrodes, the high modulus of these metals is incompatible with the flexible triboelectric layer, resulting in limited flexibility and stretchability [14]. By contrast, hydrogels, characterized by their high stretchability, biocompatibility, and eco‐friendliness, are typical soft materials with significant potential as flexible electrode materials for enhancing the performance of TENGs [15, 16]. Significantly, hydrogels also possess ionic conductivity, which stems from the combination of conductive fillers and polymer networks formed via physical and chemical cross‐linking [17]. This unique microstructure allows hydrogels to be flexibly tailored to meet the requirements of practical application scenarios. Owing to these unique advantages, hydrogels have become ideal materials for simulating human skin and have demonstrated immense application potential in wearable devices and biomedical fields [18, 19, 20].

Despite the significant potential of hydrogels across various fields, challenges remain in maintaining their performance stability under extreme environments and in improving the efficiency of their preparation. The instability of the aqueous phase within the gel network under extremely low‐temperature environments results in a considerable decrease in the mechanical and electrical properties of hydrogel‐based materials, such as TENGs [21, 22, 23]. Additionally, conventional free radical polymerization methods for preparing hydrogels often depend on extended heating or UV radiation. These processes not only require substantial energy input but also have a lengthy preparation cycle, thus hindering the large‐scale industrialized production of hydrogels [24, 25]. Therefore, there is an urgent need to develop a hydrogel preparation technique that combines rapid preparation, energy efficiency, and environmental adaptability. Recently, Li et al. [26]. pioneered an innovative approach based on a novel dual‐catalytic system composed of lignin‐modified MXene and Fe^3+^ ions. This system permits swift gelation at room temperature without external energy input. Sun et al. [27]. formulated a multifunctional conductive hydrogel (DLLMH) based on demethylated lignin‐coated liquid‐metal nanospheres (DL@LM). The preparation process employs DL@LM nanospheres as a room‐temperature free‐radical polymerization initiator, enabling rapid gelation within 280 s without external energy input. Xu et al. [28]. utilized a lignin‐Fe^3+^ autocatalytic system for the rapid gelation of hydrogels. By introducing LiCl, they significantly reduced the freezing point of the hydrogel, ensuring it retains good flexibility and electrical properties at ‐18°C. This advancement expands the application of hydrogel‐based TENGs in low‐temperature environments. However, while the rapid gelation techniques offer promise for wearable devices, the high costs of materials such as MXene, metal compounds, and LM remain a significant challenge to their widespread industrial adoption in this domain. This underscores the urgent need for the development of sustainable, efficient, and cost‐effective polymerization strategies for conductive hydrogels to address existing application challenges.

In this study, we developed a multifunctional alkali‐polyphenol (DESL‐OH^−^) synergistic self‐catalytic system that serves as the core component for initiating the copolymerization of MAA and HEA monomers. This system enables the rapid yet controllable construction of DESL/NaOH@HEA‐MAA hydrogels under ambient conditions. The efficient gelation and versatile functionalities of the hydrogels are attributed to the multiple synergistic effects between sodium hydroxide (NaOH) and lignin (DESL) with a polyphenol structure, which include: (1) NaOH facilitates and mediates dynamic redox reactions between DESL and ammonium persulfate (APS), thereby accelerating APS decomposition and subsequent generation of free radicals; (2) Hydroxide ions (OH^−^) derived from NaOH act as active intermediates that directly participate in APS decomposition, further expediting free radical polymerization via “alkali‐catalyzed heterolysis‐homolysis coupling reactions”; (3) NaOH promotes HEA monomer hydrolysis, resulting in a water‐EG binary solvent system that endows the hydrogels with exceptional frost tolerance (down to −40°C); and (4) NaOH furnishes an abundance of conductive ions, contributing to a notably high electrical conductivity (2.3–2.8 mS cm^−1^) for hydrogels. Owing to its well‐balanced properties, the hydrogel (MAA/HEA = 3:3) was selected as the flexible electrode for assembling H‐TENG. In arctic conditions of −40°C, the H‐TENG can effectively transform the mechanical energy from human compression movements into electrical energy with a maximum open‐circuit voltage (*V*_oc_) of 154 V, short‐circuit current (*I*_sc_) of 1.00 µA, and transferred charge (*Q*_sc_) of 34.6 nC. These outputs are sufficient to power multiple practical applications, such as illuminating LEDs and electronic watches, as well as enabling precise, real‐time dynamic monitoring of human motion signals. The proposed alkali‐polyphenol synergistic self‐catalytic rapid gelation system offers a novel design strategy for multifunctional hydrogel materials derived from renewable biomass. This approach offers new research perspectives and robust technical support for the development of self‐powered flexible electronic devices and intelligent human‐machine interfaces, demonstrating extensive and promising application prospects.

## 2 Results and Discussion

### 2.1 Design Principles for Rapid Gelation in Base‐Polyphenol Self‐Catalytic System

In conventional free radical polymerization of acrylic acid (AA) monomers for preparing polyacrylic acid (PAA) hydrogels, ammonium persulfate (APS) initiators typically need to be activated by external heating or UV irradiation to generate free radicals, which subsequently trigger monomer polymerization. This process is generally time‐consuming and energy intensive. More critically, the use of toxic cross‐linkers like *N,N'*‐methylene bis(acrylamide) (MBA) is often unavoidable to mediate the cross‐linking of polymer chains during gel network formation. The reliance on such cross‐linkers significantly limits the practical applications of hydrogels in fields like biomedicine and implantable electronics. To address these challenges, we developed a multifunctional alkali‐polyphenol synergistic self‐catalytic system that accelerates the free radical polymerization of target monomers at ambient temperature. This system enables rapid yet controllable gelation without external stimulation and imparts multiple functionalities, such as freezing resistance, self‐adhesion, and conductivity, to the resulting hydrogels.

**FIGURE 1:**
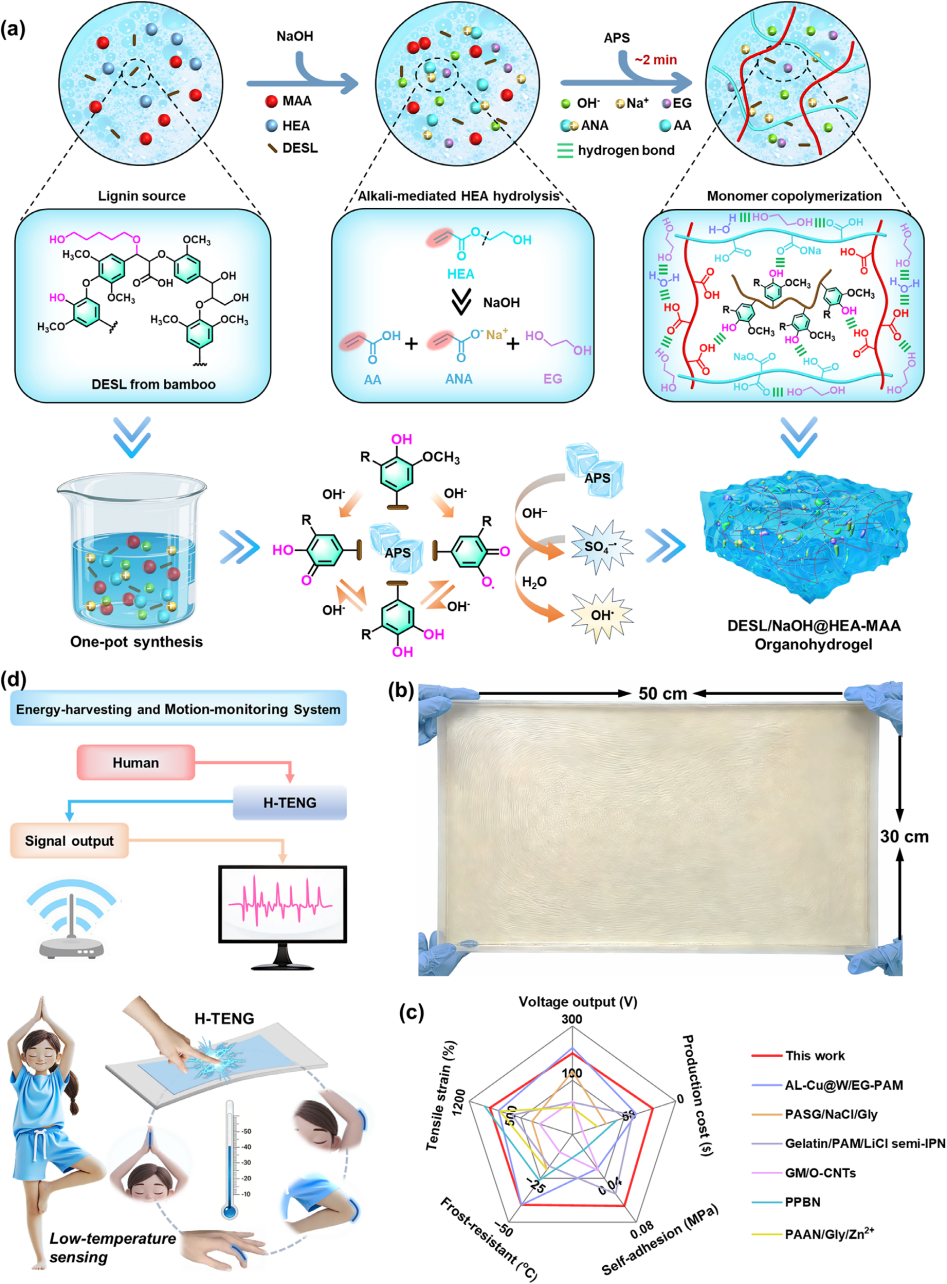
Schematic of the DESL/NaOH@HEA‐MAA hydrogels from synthesis to application. (a) Rapid polymerization process mediated by the alkali‐polyphenol synergistic self‐catalytic system. (b) Visual demonstration of the scalable, low‐cost, freeze‐resistant hydrogel. (c) Radar chart illustrating key performance metrics of the hydrogel (MAA/HEA = 3/3, DES = 0.06 wt.%) [16, 33, 34, 35, 36, 37]. (d) Application demonstration of the hydrogel in self‐powered TENGs for biomechanical energy harvesting and human motion monitoring.

In this study, a carefully designed polymerization system enables the simultaneous achievement of rapid room‐temperature gelation and frost resistance in hydrogels. The hydrogels are efficiently synthesized through a DESL‐OH^−^ synergistically driven process, which primarily involves a self‐redox reaction between DESL and APS, alkali‐catalyzed hydrolysis of HEA accompanied by in‐situ formation of sodium acrylate (ANA), and the establishment of an EG‐water binary solvent system. Figure 1a illustrates the fabrication procedure and gelation mechanism of the hydrogels. Briefly, the hydrogels are synthesized via a one‐pot approach, involving the dissolution of HEA, MAA, and DESL in a NaOH solution, followed by the addition of an APS solution. It is imperative to emphasize the multifunctional roles of the alkaline medium: (1) Owing to its strong electrolytic nature, NaOH induces the ionization of DESL and MAA, generating phenoxide anions (Ph‐O^−^) and carboxylate ions (‐COO^−^), respectively [6, 29]. These ions, in conjunction with NaOH, provide abundant conductive pathways within the hydrogels, thereby enhancing their ionic conductivity. (2) It facilitates the hydrolysis of HEA monomers, enabling the in‐situ generation of endogenous EG and ANA [30]. The reaction equation shown in Figure S1 clearly illustrates the formation pathway of endogenous EG. The synergistic interaction between the charged polar end groups (‐COO^−^) in ANA molecules and the EG‐water binary solvent bestows the hydrogels with superior low‐temperature resilience [31, 32]. (3) The Ph‐O^−^ ions derived from DESL are more electron‐rich and nucleophilic than their neutral counterparts, making them more readily oxidized by APS to form resonance‐stabilized semiquinone radicals with quinone structures [28]. The heat released from the above reactions drives APS decomposition, generating sulfate radicals (SO_4_•^−^). Critically, the synergistic combination of a moderate molecular weight (*M*_n_ = 1700 g mol^−1^, *M*_w_ = 3740 g mol^−1^) with favorable molecular uniformity (PDI = 2.21) and phenolic hydroxyl content (1.52 mmol g^−1^) is pivotal for the rapid cross‐linking. These structural attributes collectively maximize nucleophilic reactive sites while providing a stable macromolecular scaffold, fundamentally underpinning the ultrafast formation of a resilient polymer network with excellent structural integrity. (4) The OH^−^ ions directly partake in APS decomposition. Through “alkali‐catalyzed heterolysis‐homolysis coupling reactions”, more active free radicals are produced. These SO_4_•^−^ radicals react with water molecules in the alkaline solution to yield hydroxyl radicals (OH•). All radicals collaboratively initiate the radical polymerization of ANA and MAA monomers in alkaline water‐EG binary solvent, leading to rapid gelation within 2 min (Figure S2).

To further clarify the distinctive novelty of this alkali‐polyphenol synergistic strategy over previously reported alkali‐mediated systems, three advancements are highlighted: (i) Reduced initiator dosage with accelerated gelation kinetics. Conventional alkali‑mediated catalytic systems typically require a high concentration of initiator to achieve rapid polymerization. In contrast, our system leverages the redox activity of alkali‑activated DESL to generate semiquinone radicals that act as efficient co‑catalysts, significantly lowering the decomposition energy barrier of persulfate (APS). This synergistic effect enables ultrafast gelation (within 70 s) using an extremely low APS dosage, demonstrating a marked improvement in polymerization efficiency. (ii) Mitigation of polymer backbone degradation caused by high‑concentration initiator. High concentrations of APS exhibit strong oxidizing properties, which can readily induce over‑oxidation, chain scission, and degradation of the polymer backbone, ultimately compromising the mechanical performance of the resulting hydrogel. By contrast, the DESL‑mediated system facilitates gelation under mild, low‑oxidant conditions, effectively preserving the structural integrity of the polymer network and promoting the formation of a more uniform and stable 3D architecture (Figure S3). This contributes to enhanced structural stability and outstanding mechanical resilience, as evidenced by the hydrogel's exceptional tensile strain (>900%) in Figure 3c. (iii) Mild and tunable polymerization kinetics enabling synchronous functionalization. Unlike previous alkali‐mediated catalytic system that focuses solely on accelerating gelation, our synergistic catalytic strategy establishes a dynamic reaction window that concurrently supports the in‑situ hydrolysis of α‑hydroxyethyl acrylate (HEA) into ethylene glycol (EG). This unique feature endows the hydrogel with intrinsic anti‑freezing properties without the need for external cryoprotectants.

In conventional APS‑initiated systems, uncontrolled and excessively rapid polymerization tends to immobilize acrylate monomers within the crosslinked network before functional transformations (e.g., hydrolysis) can occur effectively. In contrast, the NaOH‑regulated system offers finely tuned polymerization kinetics that enable the simultaneous occurrence of radical polymerization and in‑situ hydrolysis of HEA monomers. This optimal kinetic balance ensures the endogenous formation of cryoprotectants within the hydrogel, yielding reliable mechanical and anti‑freezing performance. Consequently, a large‐scale hydrogel with dimensions of 50 cm × 30 cm rapidly fabricated via this synergistic self‐catalytic strategy exhibits a low preparation cost ($25.6) (Figure 1b,c; Tablel S2). The corresponding cost for each reagent is clearly quantified and presented in Table S3, which provides a transparent assessment of the economic feasibility and scalability of our proposed strategy. In addition, the hydrogel combines a reliable self‐adhesion strength (0.065 MPa), excellent frost resistance (sustainable performance at −40°C), and high tensile strain (918%) (Figure 1c; Table S2). When employed as the working electrode in a self‐powered triboelectric nanogenerator (H‐TENG), it not only enables efficient harvesting and conversion of biomechanical energy with a maximum stable output voltage of 200 V but also permits reliable real‐time monitoring of human motion, even under extreme low‐temperature conditions (Figure 1c,d).

**FIGURE 2:**
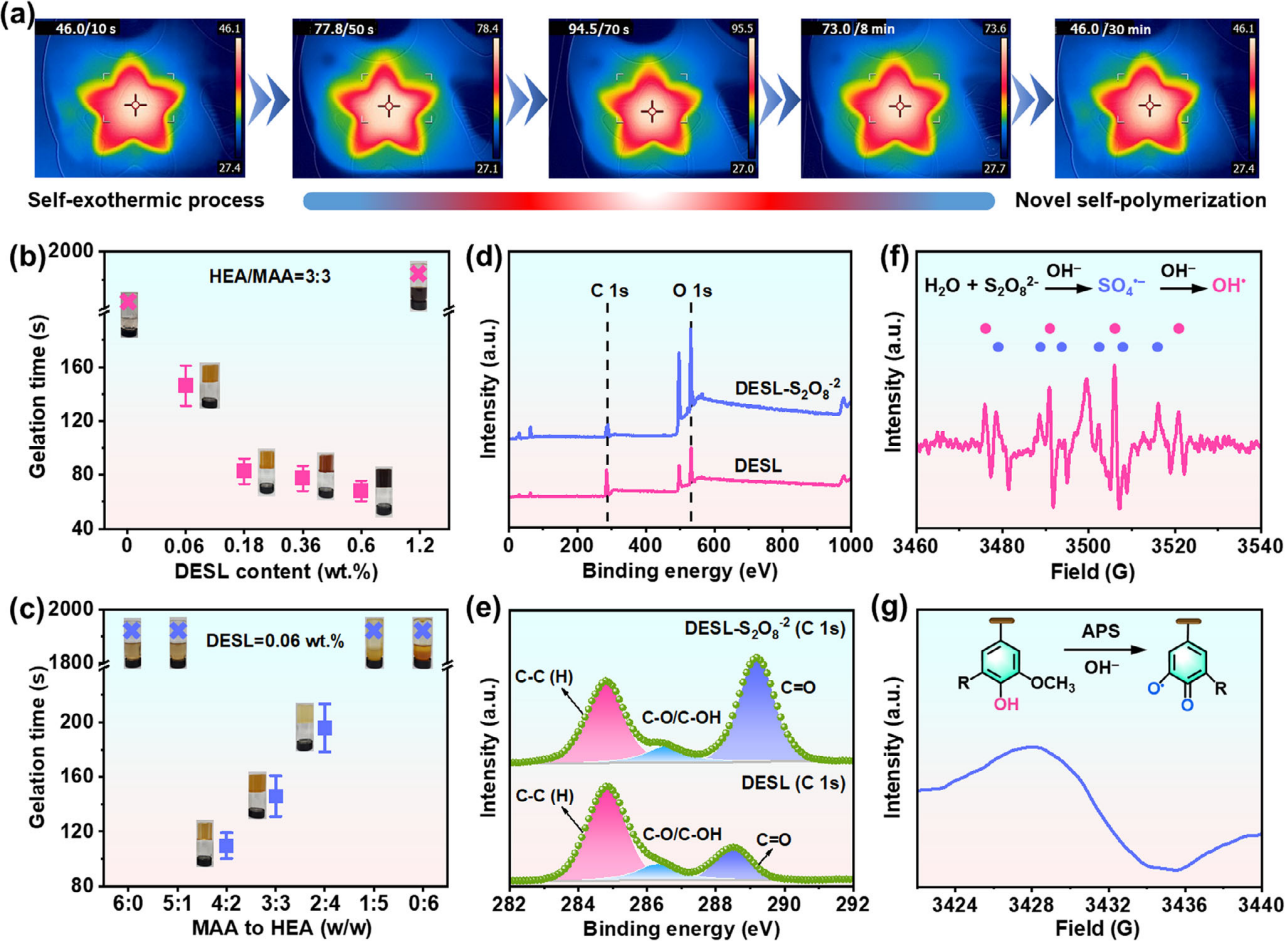
Mechanisms underlying the rapid gelation of hydrogels. (a) Exothermic evolution during room‐temperature gelation. (b, c) Gelation time vs. the DESL content and monomer mass ratios. (d, e) XPS spectra of the DESL‐APS: (d) full survey and (e) high‐resolution C 1s region. ESR spectra probing (f) hydroxyl/sulfate and (g) semiquinone radicals.

The fabricated hydrogels exhibit high transparency (>88% transmittance at 700 nm) across varying lignin contents (Figure S4). Notably, the alkali‐assisted hydrolysis of HEA, coupled with the synergistic self‐catalytic effect of DESL‐OH^−^, allows even a small amount of APS to trigger a vigorous self‐exothermic reaction within 70 s (Figure 2a), achieving rapid gelation at room temperature. To investigate the alkali‐assisted rapid gelation mechanism, control experiments without the addition of NaOH were conducted. Due to the inherent insolubility of DESL in water, DESL was subjected to sulfonation modification to improve its water solubility prior to the experiment. The results showed that the mixture without NaOH remained liquid after storage at room temperature for 6 h, with no obvious polymerization observed (Figure S5). This is in sharp contrast to the rapid gelation (within 2 min) observed in the NaOH‐containing system, strongly indicating that NaOH plays a crucial and indispensable role in initiating the gelation process.

Similarly, control experiments performed in the absence of DESL failed to form a well‐structured hydrogel even after 6 h of polymerization (Figure 2b, inset). Experimental data indicate that the gelation rate is positively correlated with the DESL content and MAA/HEA mass ratio. Specifically, increasing the DESL content from 0.06 to 0.6 wt.% reduced the gelation time from 150 to 70 s. However, a further increase to 1.2 wt.% led to gelation failure, as excess DESL scavenges free radicals and terminates chain growth [38]. Thus, DESL serves a dual role: facilitating catalysis and regulating the process by quenching excess radicals to prevent explosive polymerization. Similarly, at a fixed DESL content of 0.06 wt.%, shifting the MAA/HEA mass ratio from 2:4 to 4:2 shortened the gelation time from 196 to 109 s (Figure 2c). However, a significant imbalance in the mass ratio of the two monomers resulted in gelation failure. We attribute this to the hydrolysis of excess HEA, which consumes a substantial amount of OH^−^ ions, lowers the system pH, and thereby suppresses APS decomposition and radical generation, ultimately slowing or preventing gelation. Together, these results confirm that both lignin concentration and the monomer mass ratio are decisive factors governing the gelation rate of hydrogels in an alkaline environment.

To elucidate the mechanism underlying the rapid polymerization mediated by DESL‐OH^−^, X‐ray photoelectron spectroscopy (XPS) and electron spin resonance (ESR) spectroscopy were employed. The XPS analysis (Figure 2d,e) disclosed two characteristic peaks of DESL at 286.6 and 288.9 eV, assigned to C‐O/C‐OH and C═O, respectively [39]. The high‐resolution C 1s spectrum showed that the addition of APS to pristine DESL under alkaline conditions significantly increased the peak area corresponding to C═O. This result suggests the oxidation of hydroxyl groups (‐OH) and methoxy groups (‐OCH_3_) in DESL by APS, leading to the formation of semiquinone/quinone structures [40]. In the presence of NaOH, the ‐OCH_3_ groups within the DESL structure undergo hydrolysis, yielding highly reactive catechol structures that subsequently form catecholate salts. These salts are then oxidized by persulfate ions (S_2_O_8_^2−^) from APS to generate the semiquinone radicals necessary for initiating polymerization, as illustrated in Figure 2g. These processes concurrently promote the decomposition of APS into SO_4_•^−^ radicals, which further react with water molecules to generate OH• radicals, supplying sufficient reactive free radicals for rapid gelation. The characteristic sextet signal and 1:2:2:1 quartet signal in Figure 2f obtained from the ESR spin‐trapping tests confirm the presence of SO_4_•^−^ and OH• radicals, respectively. Based on these characterization results, it can be concluded that the DESL‐OH^−^ self‐catalytic system generates semiquinone radicals through redox reactions, while APS is reduced by DESL to form SO_4_•^−^ radicals that further reacts with water molecules to produce OH• radicals. The synergistic action of these multiple radicals enables rapid gelation at room temperature. Therefore, the proposed self‐catalytic system offers significant advantages for the rapid synthesis and precise regulation of hydrogels.

### 2.2 Mechanical and Self‐Adhesive Properties of Hydrogels

**FIGURE 3:**
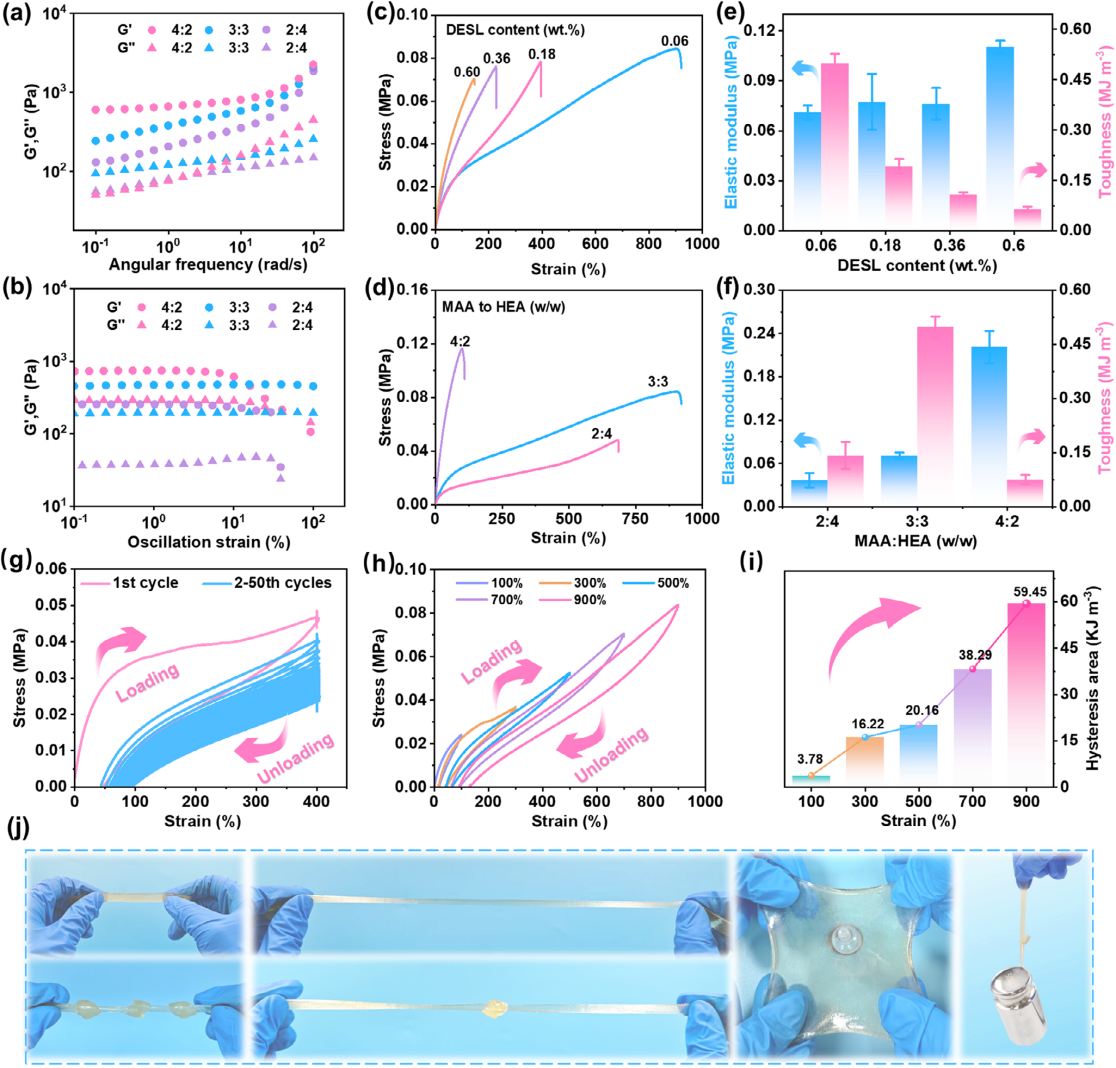
Mechanical performance of hydrogels. (a) G′ and G″ vs. angular frequency at 1% strain. (b) G′ and G″ vs. oscillation strain amplitude at 10 rad s^−1^ frequency. (c–f) Tensile strain‐stress curves, toughness, and elastic modulus (at 10% strain) of hydrogels with different DES contents (MAA/HEA = 3/3) and monomer mass ratios (DES = 0.06 wt.%). (g, h) Continuous loading‐unloading cycle curves of hydrogel (DESL = 0.06 wt.%, MAA/HEA = 3/3) at 400% strain and different strain intervals. (i) Hysteresis area under different strain cycles. (j) Mechanical performance assessed by stretching, knotting, puncturing, and lifting a 500 g weight.

Excellent elasticity, stretchability, and toughness are essential for hydrogels to be suitable for flexible electronics. Dynamic rheological tests were performed on the hydrogels to further analyze how different MAA/HEA ratios affect the modulus. Figure 3a shows the hydrogels undergoing strain scanning at 10 rad s^−1^. It was observed that the storage modulus (G′) exceeded the loss modulus (G″) for all hydrogels over the entire frequency sweep range, indicating that the gels exhibit elastic solid‐like behavior [41]. Figure 3b presents the strain sweep results of the hydrogels at an angular frequency of 10 rad s^−1^. As the proportion of MAA increases, both G′ and G″ increase accordingly. However, once the oscillatory strain exceeds a certain threshold, both G′ and G″ decrease significantly. This phenomenon is likely due to the dissociation of the cross‐linked structure within the material under large strain, which leads to the collapse of the hydrogel network. These findings are consistent with the conclusions derived from the subsequent mechanical tests.

The mechanical properties of the hydrogels with different DESL contents and monomer compositions were further evaluated using a tensile testing system. As shown in Figure 3c and Figure S6a, the tensile strain of the hydrogels increased significantly as the DESL content decreased, reaching up to 918% when the DESL content was 0.06 wt.%. This result indicates that a lower DESL content effectively enhances the flexibility and ductility of the hydrogels, which could be attributed to the hydrogen‐bond interactions between the MAA/HEA copolymer and DESL. However, the tensile stress decreased steadily from 0.084 to 0.069 MPa with increasing DESL content from 0.06 to 0.6 wt.%. The toughness of the DESL/NaOH@HEA‐MAA hydrogels shows a similar trend, while the elastic modulus shows the opposite trend. When the DESL ratio was fixed at 0.06 wt.%, the maximum toughness and elastic modulus reached 0.50 MJ m^−3^ and 0.071 MPa, respectively. This trend can be attributed to two factors. First, DESL scavenges certain free radicals, thereby inhibiting monomer polymerization, which reduces the cross‐linking density of the gels and leads to a significant decrease in tensile strength. Second, the weakening effect caused by the formation of DESL aggregates on the hydrogel surface is further exacerbated [42]. Therefore, fixing the DESL content at 0.06 wt.% conferred the hydrogels with relatively high mechanical strength (Figure 3e), and this content was used in subsequent studies investigating the effect of monomer mass ratio on mechanical properties.

As revealed in Figure 3d,f and Figure S6b, the hydrogels exhibited balanced mechanical properties when the MAA/HEA mass ratio was 3:3. Specifically, HEA hydrolyzes under alkaline conditions to produce EG and sodium acrylate. The ‐OH groups in the EG molecules can form multiple hydrogen bonds with the carboxyl groups (‐COOH) on the polymer chains, which fix EG molecules within the gel network and increase the number of crosslinking points and interactions, thereby enhancing the stability and regularity of the gel network. This improvement in mechanical properties includes enhanced tensile strain and toughness. Additionally, the ability of hydrogen bonds to repeatedly break and reform is crucial for the gel's self‐healing capability. When the gel is damaged by external forces, its internal hydrogen‐bond network is disrupted. However, due to the dynamic reversibility of hydrogen bonds, under appropriate conditions, hydrogen bonds will reform, restoring the gel's structure and properties. Interestingly, the stress‐strain curve of the hydrogels exhibits nonlinear mechanical behavior (Figure S6c) and can be divided into two regions. In the small strain range (0%–100%, Region I), the initial modulus primarily depends on the degree of hydrogen‐bond cross‐linking, and stress increases rapidly with strain during this stage. In the larger strain range (100%–910%, Region II), continuous stretching causes hydrogen bonds dissociation, leading to a slow increase in stress with strain and thereby dissipating mechanical energy. The overall curve reflects the distinct mechanical response of the hydrogel at different strain stages, which is mediated by hydrogen bonding.

To further evaluate the fatigue resistance performance of the hydrogels, continuous cyclic tensile tests (400% strain) were conducted on the hydrogel containing 0.06 wt.% DESL with a MAA/HEA mass ratio of 3:3. As illustrated in Figure 3g, the hydrogel exhibits excellent fatigue resistance. After 400% deformation, significant hysteresis was observed during the first unloading cycle. This indicates that the hydrogel dissipated a substantial amount of energy through multiple internal deformations, which helps delay its complete fracture [43]. From the second to the 50th consecutive loading‐unloading cycle tests, the stress‐strain curve remained intact, with the maximum stress retaining over 51% of its initial value (Figure S6d). This behavior is attributed to the relaxation of reversible hydrogen bonds, confirming that the hydrogel has excellent deformation recovery capability and fatigue resistance. Additionally, tensile loading‐unloading tests were performed at strains ranging from 100% to 900% to investigate the hydrogel's energy dissipation behavior (Figure 3h). The results show that a larger hysteresis loop area corresponds to higher energy dissipation efficiency. Figure 3i shows that the energy dissipated per unit volume was positively correlated with strain amplitude, highlighting the gel's ability to achieve efficient strain energy dissipation through the disruption of its dynamic hydrogen‐bond network. In addition to the high strength, the hydrogel's mechanical robustness is confirmed by its reliable foldability and stretchability (Figure 3j).

**FIGURE 4:**
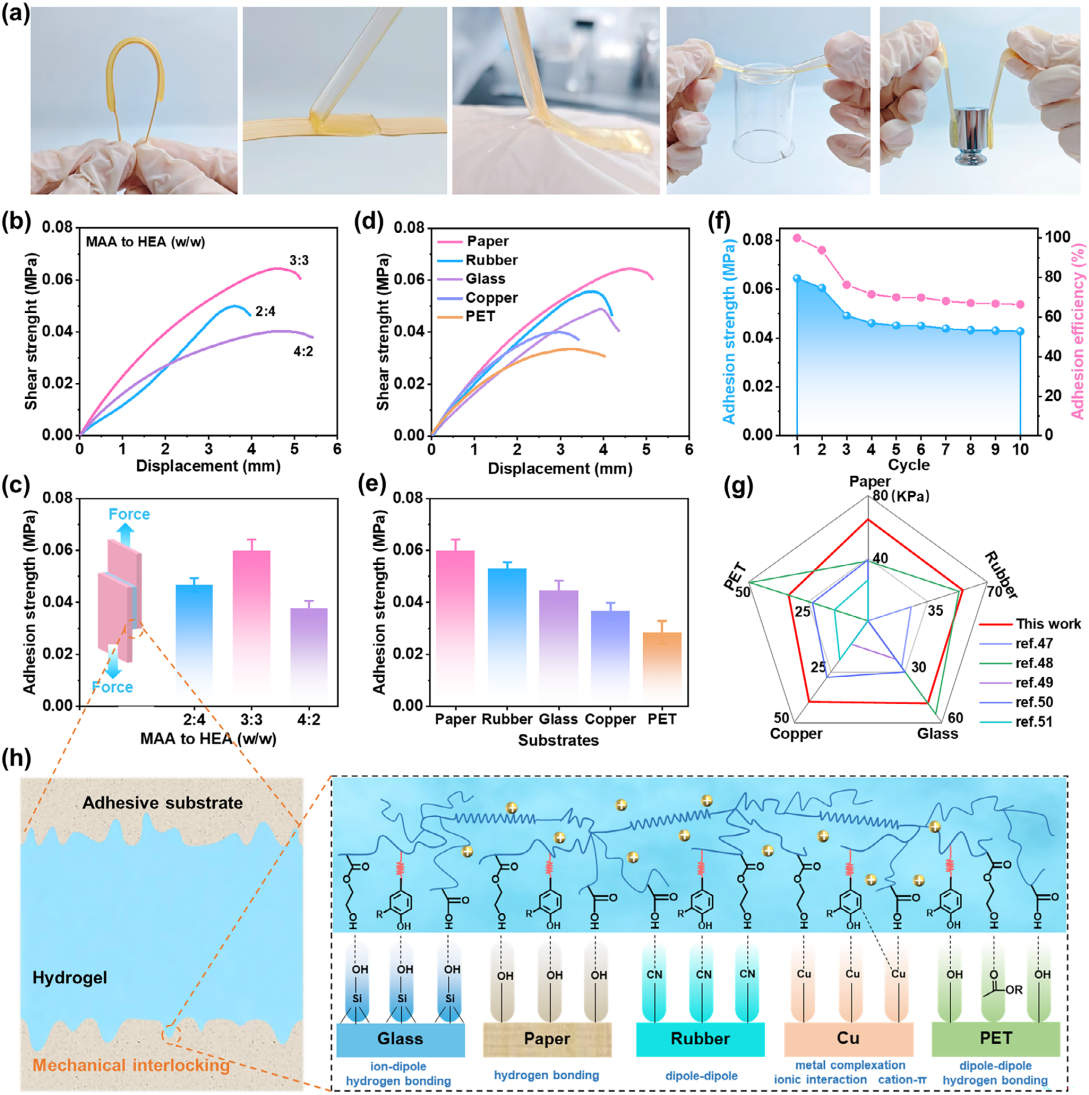
Self‐adhesion properties of hydrogels. (a) Images of hydrogel adhering to different substrate surfaces. (b, c) Adhesive shear force vs. displacement of hydrogels with different monomer mass ratios and the corresponding adhesion strengths. (d, e) Adhesive shear force vs. displacement of the hydrogel on different substrates and the corresponding adhesion strengths. (f) Adhesion strengths and efficiencies of hydrogel (MAA/HEA = 3/3) on paper surfaces after 10 adhesion cycles. (g) Comparative adhesion graph [47, 48, 49, 50, 51]. (h) Adhesion mechanism schematic of hydrogel on four typical substrates, where R represents ‐OCH_3_ groups.

A distinctive feature of our lignin‐based hydrogels lies in their intrinsic adhesiveness toward diverse substrates. This property effectively prevents interfacial delamination in hydrogel‐based TENGs while enhancing signal stability through sustained comfort and intimate interfacial contact, thereby substantially improving operational efficiency. The as‐prepared hydrogels demonstrate durable and repeatable adhesion across various surfaces including paper, plastic, metal, stone, wood, foam, glass, skin, and rubber, as evidenced by Figure 4a and Figure S7. They also exhibit robust adhesion to human fingers, maintain prolonged adhesion on tissues with adaptive conformability, and allow residue‐free removal. No irritation or allergic reactions were observed during adhesion testing, substantiating their excellent biocompatibility. Considering the critical dependence of monomer composition on self‐adhesive performance, lap shear strength tests were systematically conducted to evaluate the adhesion characteristics of hydrogels with varying monomer ratios on paper substrates. As presented in Figure 4b,c, the adhesion strength exhibits a trend of first increasing and then decreasing with elevated MAA/HEA ratio.

This enhancement likely originates from the increased generation of in‐situ formed EG during HEA hydrolysis, which strengthens interfacial bonding through two primary mechanisms: First, EG's higher viscosity compared to water optimizes interfacial wettability between hydrogel and substrate; second, EG forms stronger interactions with substrate surfaces than water molecules, enabling more stable interfacial bonding [44]. However, excessive EG concentration diminishes adhesion, potentially due to an overdeveloped hydrogen bonding network that creates high‐strength cohesive forces within the hydrogel matrix, leading to decreased adhesive strength. To identify the optimal conditions, the adhesive strength of hydrogel (MAA/HEA = 3/3) on copper, glass, PET, paper, and synthetic rubber was analyzed (Figure 4d,e). Among all tested substrates, the hydrogel showed the highest adhesion strength on paper (0.065 MPa), followed by glass (0.049 MPa), copper (0.040 MPa), and PET (0.034 MPa) (Figure 4e). The adhesion of hydrogel to various substrates originates from ‐COOH groups in polymer chains and the incorporation of DESL (containing catechol groups), EG (rich in ‐OH groups), and metal ions introduced during synthesis process. These components interact with molecules of substrate through non‐covalent bonds such as hydrogen bonds, π–π stacking, and cation–π interactions [45, 46]. For instance, paper, which is mainly composed of cellulose, offers a high density of binding sites for ‐OH groups, enabling strong adhesion via extensive hydrogen bonding. As shown in Figure 4f, both the adhesive strength and efficiency of the hydrogel progressively declined over repeated adhesion‐separation cycles, suggesting a gradual degradation in its adhesive capacity due to repeated interfacial contacts. After 10 cycles, the decline rate diminished and the adhesion performance stabilized, with a retained shear strength on paper of 0.043 MPa. These results indicate that, although the adhesion properties were partially compromised, the hydrogel still maintained a degree of adhesion, confirming its certain reusability.

The adhesion of hydrogels originates from the multi‐mechanism interactions between functional groups in their molecular structure and substrate surfaces. The adhesion mechanism involves the functional groups in hydrogels (such as ‐COOH and ‐OH groups) forming complex interfacial interactions with different substrates, including hydrogen bonding, ion‐dipole interaction, metal coordination, cation‐π interaction, and electrostatic interactions, as illustrated in Figure 4h. Such a multi‐modal adhesion mechanism endows the hydrogels to form stable and durable interfacial bonds with various material surfaces. Moreover, compared with the adhesive strength of copolymerized gels reported in recently published literatures (Figure 4g), the fabricated hydrogels in this work show an overwhelming advantage [47, 48, 49, 50, 51]. These results confirm that the lignin‐based hydrogels possess excellent adhesive properties, meeting the fundamental requirements for integrated applications in TENG‐related soft electronics.

### 2.3 Frost Resistance and Electrical Conductivity of Hydrogels

**FIGURE 5:**
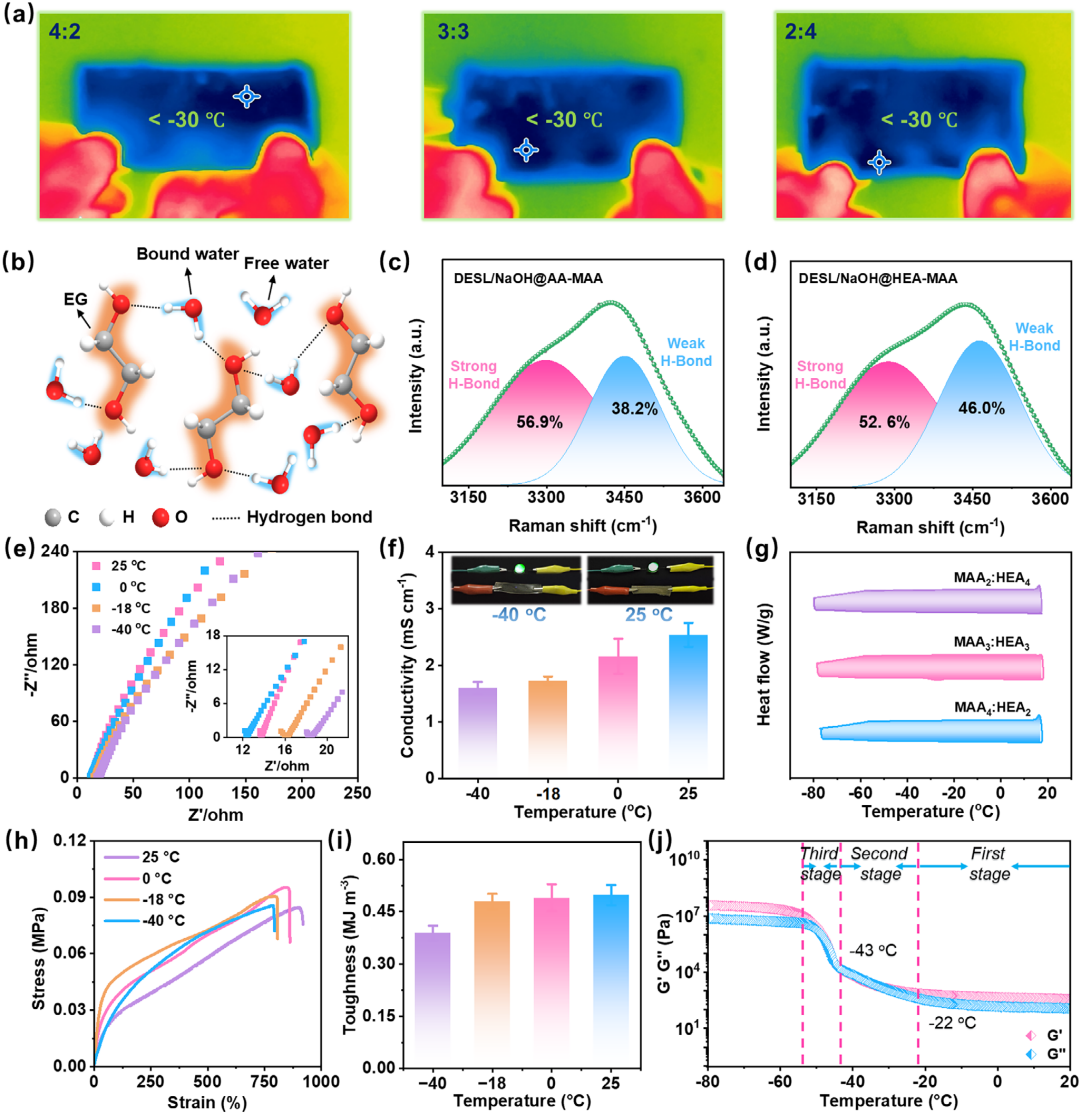
Extreme environment adaptability of hydrogels. (a) Thermal imaging of the folded gels with different monomer ratios after −40°C freezing. (b) Diagram of EG‐water hydrogen‐bond formation mechanism. (c, d) Raman spectra and the corresponding curve‐fitting of the hydrogel in the O‐H stretching region reveal the fitted sub‐bands assigned to water molecules with strong and weak hydrogen bonds. (e, f) Electrochemical impedance spectra and conductivities of the hydrogel (MAA/HEA = 3/3) under different temperatures of freezing. Inserts: photographs of LED lit up by the gel acting as a conductor at different temperatures. (g) DSC curves. (h, i) Tensile stress‐strain curves and toughness of the hydrogel after refrigeration at different temperature gradients. (j) Rheological temperature sweep spectra of the hydrogel measured at a constant frequency of 10 rad s^−1^ and a strain of 1%.

It is worth noting that the hydrogels developed in this study demonstrate exceptional anti‐freezing properties and electrical conductivity, thus enabling stable operation of the resulting flexible devices such as TENGs under cryogenic conditions. Thermal imaging provides visual confirmation of the cryogenic tolerance of hydrogels (Figure 5a). Following exposure to subzero conditions at −40°C for 6 h, the materials preserved their inherent flexibility and ductility. The freezing of water is essentially a hydrogen‐bond‐mediated phase transition from disordered water molecules to an ordered ice crystal lattice [52]. Conventional hydrogel networks contain abundant free water molecules that readily undergo hydrogen‐bond rearrangement and form ordered ice crystals at subzero temperatures. This structural transformation compromises the mechanical flexibility and stretchability of hydrogels, restricting their application in the construction of wearable electronic devices. In our design, HEA monomer was strategically introduced during hydrogel synthesis. The EG generated through HEA hydrolysis competes with inter‐water hydrogen bonds to establish novel EG‐water hydrogen bonding networks (Figure 5b). This molecular interaction effectively disrupts the ordered arrangement of water molecules, inhibits the growth of ice crystals, reduces the freezing point of hydrogels, and substantially improves their low‐temperature operational stability [53].

To elucidate the underlying mechanism, we utilized Raman spectroscopy to analyze the hydrogen‐bond interactions between adjacent water molecules/water and EG. Gaussian‐Lorentzian deconvolution was employed to systematically resolve the broad O‐H stretching envelope (3100‐3800 cm^−1^) into two characteristic sub‐bands, each assigned to a distinct hydrogen‐bonding environment. Specifically, the high‐frequency component at approximately 3450 cm^−1^ signifies a population of water clusters with localized, weak hydrogen bonds. Conversely, the spectral component shifted toward 3250 cm^−1^ denotes the presence of a highly ordered, strongly hydrogen‐bonded water fraction (Figure 5c,d) [33, 54]. Compared with the hydrogel (DESL/NaOH@AA‐MAA) prepared by copolymerization of AA and MAA monomers, the characteristic peak detected at 3450 cm^−1^ for DESL/NaOH@HEA‐MAA organohydrogel shifted to 3444 cm^−1^, and the ratio of strong to weak hydrogen bonds decreased (Figure S8). This phenomenon indicates that the strong hydrogen‐bond interactions between water molecules in the DESL/NaOH@HEA‐MAA organohydrogel are weakened by the synergistic effect of the EG‐water binary solvent system, which hinders water molecules from forming stable ice crystal structures, thus enhancing the frost resistance of hydrogels. To systematically evaluate the influence of monomer composition on phase transition characteristics, differential scanning calorimetry (DSC) analyses were conducted for hydrogels (Figure 5g). The absence of distinct ice crystallization peaks in the thermograms demonstrates that EG incorporation effectively suppresses ice nucleation across the operational temperature range of −80°C to ambient conditions, thereby establishing robust anti‐freezing properties within this interval. Mechanical characterization presented in Figure 5h,i reveals consistent stress‐strain behavior of the hydrogel with a MAA/HEA mass ratio of 3:3 across different temperature intervals. Both fracture elongation and maximum tensile strength were largely preserved over the wide range of −40–25°C. These findings validate the hydrogel's capacity to sustain mechanical integrity under cryogenic conditions.

Rheological investigations were further performed to elucidate the freeze‐resistant mechanisms. As shown in Figure 5j, both the G' and G'' exhibit three distinct stages as a function of temperature. Initially, they remain stable during cooling from 20°C to −22°C without phase transition events [55]. In the second stage, gradual modulus enhancement occurs from −22°C to −43°C, attributed to strengthened intermolecular interactions and restricted polymer chain mobility [56]. In the third stage, the hydrogel exhibits a glass transition temperature of approximately −43°C; below this threshold, it becomes brittle due to complete vitrification [57]. Therefore, the hydrogels fabricated in this work maintain relative flexibility even at temperatures as low as −43°C, thus endowing them with a broad applicable temperature range. The comparison data in Figure 1c clearly demonstrate that the hydrogel prepared in this study exhibits one of the highest levels of cryo‐tolerance among recently reported state‑of‑the‑art anti‑freezing hydrogels. Most conventional anti‐freezing hydrogels depend on the external introduction of cryoprotectants or involve complicated multi‐step fabrication procedures. In sharp contrast, our hydrogel realizes intrinsic anti‐freezing performance via the in‐situ generation of EG under mild and controllable polymerization conditions, thus avoiding any external cryoprotectants. This novel and facile strategy not only simplifies the manufacturing process but also addresses typical drawbacks of conventional systems, including cryoprotectant leakage, phase separation, and poor environmental compatibility. Furthermore, our system achieves an exceptional combination among ultrafast ambient gelation (∼2 min), extreme low‑temperature functionality (−40°C), and superior cost‑effectiveness ($0.017 cm^−2^).

Additionally, the electrical conductivity of hydrogels at low temperatures with different monomer ratios was investigated. The incorporation of NaOH endows the hydrogels with high ionic conductivity because this strong electrolyte creates an alkaline environment that promotes the ionization of phenolic ‐OH groups in DESL and ‐COOH groups in MAA to generate negatively charged species such as ‐O^−^ and ‐COO^−^ while releasing free Na^+^ ions. These Na^+^ ions as primary charge carriers not only migrate freely within the pores of the hydrogel's three‐dimensional network but also move along the polymer chains through electrostatic interactions with the charged groups. These ionic transport behaviors directly contribute to high ionic conductivity of the hydrogels. It can be observed from Figure S9a,b that the impedance of hydrogels with different monomer compositions ranged from 6 to 14 Ω, and the conductivity decreased accordingly from 3.4 to 2.1 mS cm^−1^. This decrease in conductivity with increasing proportion of HEA monomers could be attributed to EG, a product of HEA hydrolysis. While EG acts as an anti‐freeze, it may also increase the tortuosity of the ion transport path or reduce the number of effective charge carriers. The temperature dependence of the hydrogel's electrical properties was also investigated. As shown in Figure 5e, when the storage temperature was reduced from 25°C to −40°C, the electrical resistance of the hydrogel increased gradually from 12 to 18 Ω, and the conductivity decreased accordingly from 2.5 mS cm^−1^ to 1.6 mS cm^−1^. This reduction in conductivity is likely due to the slowing down of ion migration within the gel network at low temperatures [58]. The inset in Figure 5f demonstrates that the frozen hydrogel can act as a conductor to successfully light up LED at different temperatures, further confirming its excellent anti‐freezing ability. These results fully verify that all hydrogels possess excellent freeze resistance and electrical conductivity, indicating their potential for low‐temperature applications.

### 2.4 Operating Principle and Electrical Output Performance of H‐TENG

**FIGURE 6:**
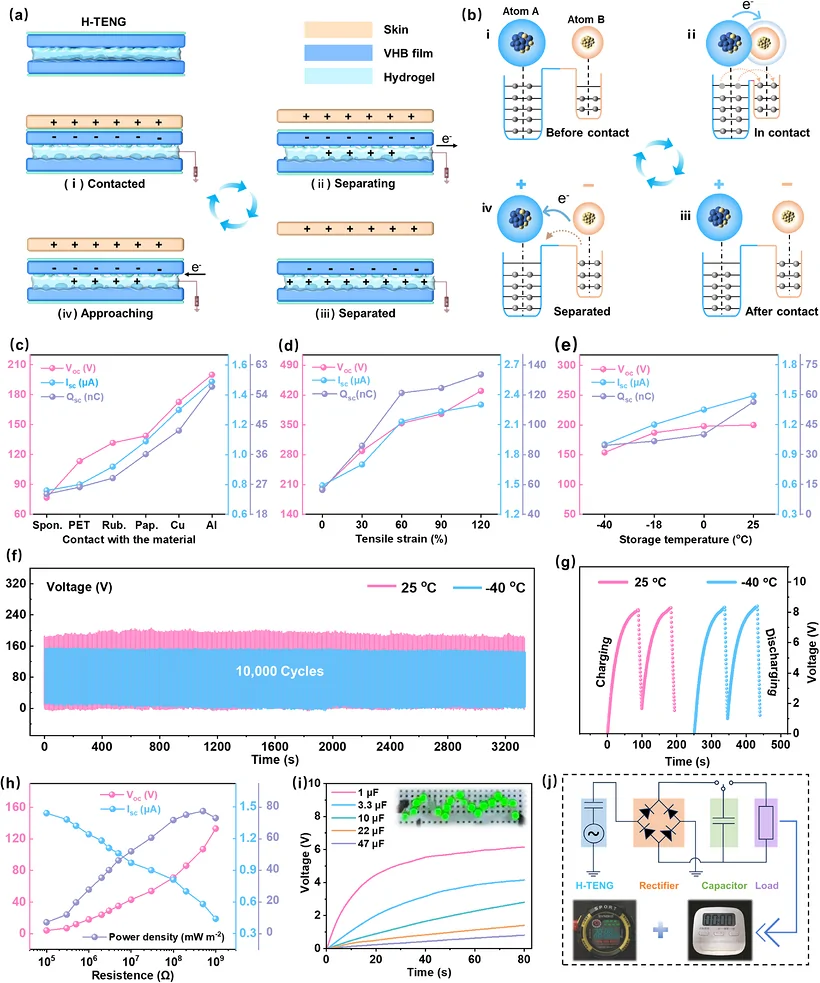
Working principle and electrical output performance of H‐TENG. (a) Schematic of the single‐electrode working principle of H‐TENG. (b) Schematic illustrating electron transfer between electron‐donating and electron‐accepting atoms. *V*_oc_, *I*_sc_, and *Q*_sc_ of H‐TENG (c) contacted with different materials, (d) under different tensile strains, and (e) at different storage temperatures. (f) *V*_oc_ stability of H‐TENG stored at 25°C and −40°C, respectively. (g) Charge‐discharge curves of H‐TENG at 25°C and −40°C, respectively. (h) *V*_oc_, *I*_sc_, and power density of H‐TENG with different external resistances. (i) Charging curves of H‐TENG for capacitors with different capacitances. Insets: photographs of H‐TENG powering LEDs light. (j) Circuit diagram of the H‐TENG self‐charging power system and a photograph demonstrating its capability to sustainably power an electronic watch.

Based on the above results, the as‐prepared hydrogel (MAA/HEA = 3/3), possessing suitable mechanical flexibility, self‐adhesiveness, frost resistance, and conductivity, is well‐suited for energy harvesting devices and flexible/wearable electronics, which thus enables a successful construction of contact‐separation mode triboelectric nanogenerator (H‐TENG) using the hydrogel as electrode material (Figure 6). The H‐TENG is mainly composed of two parts, a triboelectric layer (VHB) responsible for charge induction and an electrode layer (hydrogel) that facilitates charge transfer [59]. Figure 6a illustrates the operating principle of the H‐TENG in a single‐electrode mode, clarifying the mechanism of charge generation underlying the synergistic effects of triboelectrification and electrostatic induction [60]. Specifically, in the initial operating state (stage i), when the contact material (e.g., copper sheet, human skin) having a lower electron affinity than the VHB film interacts with the H‐TENG, an equivalent oppositely charged surface will form based on the triboelectric effect. Subsequently, as the contact material separates from the H‐TENG (stage ii), the charge separation creates a potential difference. Equal amounts of opposite charges accumulate on the surfaces of the VHB elastomer and the hydrogel, respectively. To balance this charge difference, electrons flow through the external circuit, generating a current. At the maximum separation distance (stage iii), the charge accumulation reaches a certain level, and the interfacial electric field is stabilized. When the contact material and H‐TENG approach each other again (stage iv), the electrons in the external circuit flow in the reverse direction, and the system gradually returns to a state close to the initial contact. This cyclic contact‐separation process enables the effective conversion of mechanical energy into electricity by leveraging periodic charge separation and recombination [61, 62].

What needs to be emphasized is that the triboelectric charging process exhibits inherent subtlety and complexity. To elucidate its microscopic mechanism, the electron cloud‐potential well model was employed. This theoretical framework posits that charge transfer originates from disparities in electron affinity between two interacting materials, resulting in one material becoming positively charged and the other becoming negatively charged [63]. As illustrated in Figure 6b, the charge transfer phenomenon can be systematically analyzed through four distinct stages: pre‐contact, contact, post‐contact, and separation. Prior to physical interaction (stage i), the constituent atoms of the two materials maintain independent electron cloud distributions with stable potentials, which well confine the electrons and prevent them from escaping freely, thereby preserving electrical neutrality. When the atoms approach and contact each other (stage ii), electron migration initiates the triboelectric charging process, driven by differences in electron binding energy (also termed electron affinity). Atoms with higher electron affinity capture electrons from those with lower affinity, accompanied by mutual penetration and hybridization of their electron clouds. Prolonged contact allows the system to achieve a dynamic equilibrium state (stage iii), where the surface charge density stabilizes. Upon material separation (stage iv), the previously transferred electrons make the atoms retain residual charges, thereby completing the transition from electrical neutrality to a charged state. To quantitatively verify this model, we also simulated the potential distribution of the VHB film and triboelectric material during interactive motion using COMSOL software (Figure S10a). These simulations demonstrate how the expected potential variation of the H‐TENG at different spacer distances drives electron flow to balance the potential difference (maintain electrostatic equilibrium), thereby revealing the power generation principle and operational efficiency of the H‐TENG. As the core conductive layer in the sandwich structure, the hydrogel electrode acts as a key medium for charge induction and transport. Its high ionic conductivity offers abundant mobile ions and rapid migration pathways, which significantly lower the internal impedance of the device (Figure S11). This facilitates efficient charge transfer and rapid electrostatic induction at the hydrogel‐VHB interface, ensuring stable and sufficient charge separation during repeated contact‐separation cycles. Therefore, the high ionic conductivity of the hydrogel electrode is indispensable for achieving superior electrical output in the H‐TENG.

Figure S10b,c shows the typical time‐dependent *V*_oc_ and *I*_sc_ curves generated by the H‐TENG, respectively, which reflect its dynamic electrical output characteristics during the contact‐separation. It is worth noting that the triboelectric process is synergistically dominated by both the intrinsic properties of contact materials and external environmental factors. A practical single‐electrode TENG should be capable of distinguishing contact materials with different intrinsic properties. To investigate the influence of the intrinsic properties of triboelectric materials on electrical output performance of the H‐TENG, we quantitatively analyzed the *V*_oc_, *I*_sc_, and *Q*_sc_ generated by pressing with various contact materials including sponge, rubber, PET, paper, copper (Cu) and aluminum (Al). It can be found from Figure 6c and Figure S12a–c that both *V*_oc_, *I*_sc_ and *Q*_sc_ of the six materials follow the order: Al>Cu>paper>rubber>PET>sponge. The stable *V*_oc_, *I*_sc_, and *Q*_sc_ for the Al material reached about 200 V, 1.49 µA, and 56.4 nC, respectively [16]. In view of the optimal electrical output capability, Al material was selected as the contact material to further study the impacts of external factors such as hydrogel electrode area and thickness as well as the VHB elastomer thickness on the electrical output. As detailed in Figure S13a–c, increasing the gel area could effectively promote charge accumulation and enhance interfacial charge transfer efficiency, thereby improving the electrical output of H‐TENG. Moreover, a slight upward trend in the *V*_oc_, *I*_sc_, and *Q*_sc_ of the H‐TENG was observed as the electrode thickness decreased from 4 to 1 mm. This phenomenon is primarily attributed to the improved interfacial charge transfer efficiency by reducing effective separation distance between the frictionally charged materials and strengthened electrostatic induction effect (Figure S13d–f). Meanwhile, the electrical output performance of the H‐TENG during different stretching processes was also evaluated. The measured *V*_oc_, *I*_sc_, and *Q*_sc_ were about 200 V, 1.49 µA, and 56.4 nC at the initial state, respectively, which boosted with increasing strain levels (Figure 6d; Figure S13g–i). At a strain level of 120%, the peak *V*_oc_, *I*_sc_, and *Q*_sc_ reached 430 V, 2.3 µA, and 133.8 nC, respectively. It is worth noting that these variations are mainly due to the coupling effect of reduced dielectric layer thickness and augmented contact surface area. Based on the Poisson effect, the dielectric layer became narrower under applied strains. This shortened the distance between surface charges and hydrogel electrodes, enhancing interfacial charge interactions. In addition, the increase of the contact surface area caused by the stretching of the H‐TENG is favorable for more charge transfer, which ultimately leads to a corresponding increase in the electrical output performance.

The H‐TENG can efficiently harvest mechanical energy and converts it into electrical signals, delivering stable, robust, and environment‐tolerant electrical performance over long‐term contact‐separation motion cycles across a wide temperature range (−40–25°C). As depicted in Figure 6e and Figure S14a–c, the H‐TENG achieved *V*_oc_ values of 200, 198, 187, and 154 V at 25, 0, −18, and −40°C, respectively. Even at −40°C, it retained a *V*_oc_ of 154 V, an *I*_sc_ of 1.00 µA, and a *Q*_sc_ of 34.6 nC, with merely a marginal attenuation in electrical output. At subzero temperatures, adsorbed water molecules on the surface of the H‐TENG inevitably form an ice crystal layer, thereby impairing the charge transfer efficiency. Therefore, the slight reduction in electrical output at low temperatures originates from its sensitivity to the effective contact area between the VHB film and the triboelectric layer, a key factor noted earlier. Furthermore, when subjected to 10 000 consecutive mechanical cycles at 25°C and −40°C, the H‐TENG retained consistent *V*_oc_ values of 200 and 154 V, respectively, underscoring its long‐term operational stability and structural integrity even in polar regions. (Figure 6f). Notably, we developed a self‐charging power system that employs a bridge rectifier to convert the collected alternating current (AC) into direct current (DC), capable of directly driving commercial smartwatches without complex power management circuits (Figure 6j). This system also demonstrates reliable low‐temperature operation, with corresponding performance data summarized in Figure 6g. Whether at 25°C or −40°C, the H‐TENG rapidly charges a 22.0 µF capacitor to 8.0 V. This underscores the potential of the H‐TENG as a robust energy‐harvesting platform, establishing it as a pragmatic solution to the energy autonomy challenges faced by wearable electronics in extreme environments.

To evaluate the output power of the H‐TENG in practical application scenarios, a variable resistor was integrated into the system as an external load and the power output characteristics were systematically investigated by dynamically adjusting the load resistance. As shown in Figure 6h, when the external resistance increased from 10^5^ to 10^9^ Ω in the H‐TENG system, the corresponding output voltage increases accordingly, which is consistent with Ohm's law. Meanwhile, the output current decreased accordingly, verifying that there is a negative correlation between voltage and current. Impressively, the H‐TENG exhibited a maximum power density of 77.6 mW m^−2^ with an external resistance of 500 MΩ. Additionally, to further verify its practical value as a wearable energy source, the charging characteristics of the energy storage components with different capacitance values were also systematically tested (Figure 6i). The experimental results showed that the H‐TENG could rapidly charge commercial energy storage capacitors ranging from 1 to 47 µF under constant mechanical excitation, and the output voltage ranged from 0.6 to 6.9 V. More intuitive demonstrations showed that the H‐TENG could power 18 series‐connected LEDs, which emitted light upon simple mechanical pressing, highlighting its excellent energy conversion efficiency. In addition, following 60 s of manual tapping for energy harvesting and storage, it could continuously power a smartwatch or similar electronic device for over 20 s (Video S2). These experimental results collectively confirm the application potential of the H‐TENG in powering micro‐power electronic devices.

### 2.5 H‐TENG for Self‐Driven Sensing Research

**FIGURE 7:**
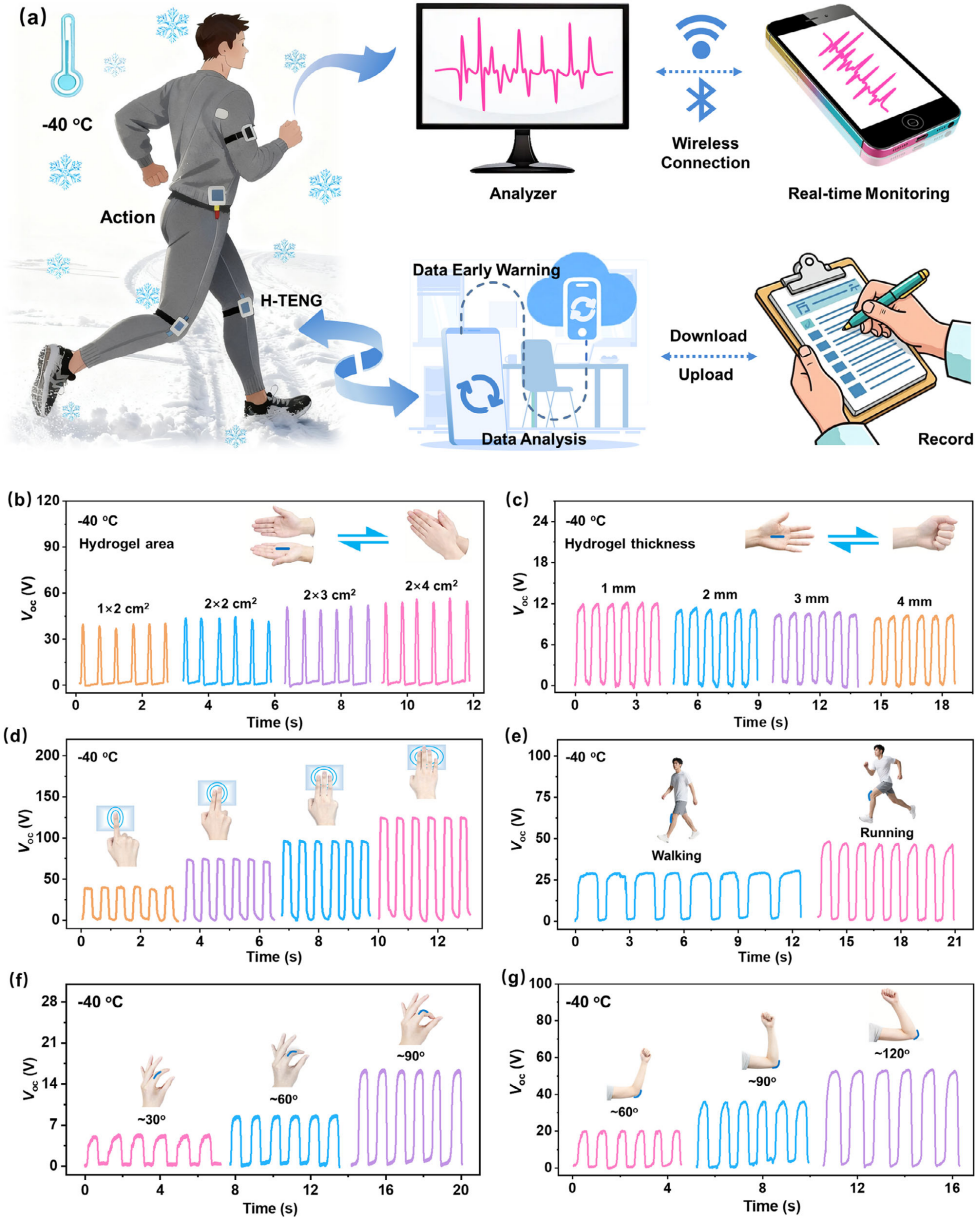
Performance of the self‐powered H‐TENG‐based human motion monitoring system. (a) System Schematic of the self‐powered H‐TENG sensor for human motion detection and human‐computer interaction in a −40°C environment. Demonstrations and output voltages of (b) clapping, (c) folding, (d) finger tapping, (e) walking and running, (f) finger bending, and (g) elbow motions at −40°C.

Given the close relationship between human movement and physical health, accurately monitoring limb movement parameters, such as step count and frequency, is of significant value for health management. Such monitoring aids in quantifying energy expenditure, enables scientific health assessments, and guides individuals in establishing regular exercise habits through data feedback. To conclude, we developed the H‐TENG integrated with the soft hydrogel electrode, which endows the device with exceptional flexibility, freezing resistance, and conductivity, establishing a foundation for real‐time human motion tracking and pressure detection. The schematic of Figure 7a depicts that the self‐powered H‐TENG can be configured as a human motion monitoring system capable of reliably and effectively converting various mechanical stimuli into analyzable electrical signals based on the coupling effect of contact electrification and electrostatic induction, without requiring an external power source, even in extreme low‐temperature environments as low as −40°C. When deformed by an external mechanical stimulus, the device undergoes a change in the contact area or separation distance between its triboelectric layers, thereby generating a corresponding electrical output signal. This capability enables the H‐TENG to be widely used in multiple fields, including wearable health monitors, tactile electronic skin, and human‐machine interfaces.

During human movement, the device captures motion‐related data and transmits it wirelessly via Bluetooth to a computer or smartphone for real‐time monitoring and analysis. Simultaneously, the data can also be uploaded to the cloud for retrospective analysis, enabling the evaluation of specific motion patterns and the maintenance of detailed records. This system thus demonstrates the H‐TENG's ability to monitor human motion, transmit signals wirelessly, and support multi‐terminal real‐time tracking even under harsh low‐temperature conditions. By harvesting mechanical energy from daily activities (e.g., walking and running) and converting it into electrical signals, the H‐TENG exhibits significant potential for self‐powered sensing applications. To validate the sensing performance of the H‐TENG, we performed a series of sensing tests on the device at −40°C. Initially, it was attached to the palm to conduct clapping and bending tests (Figure 7b,c). Tests on H‐TENGs with gel electrodes of different dimensions demonstrated that the *V*_oc_ increases with increasing electrode area but decreases with increasing electrode thickness. This is because a larger contact area enhances triboelectric charge generation, while increased thickness reduces the efficiency of interfacial charge sensing.

As shown in Figure 7d, the H‐TENG efficiently harvests energy from finger taps of varying intensities and converts it into voltage signals. In addition to simple tapping motions, this capability can be further extended to monitor complex human movements (e.g., joint bending), illustrating the versatility of the device in diverse application scenarios (Figure 7e–g). As presented in Figure 7e, the magnitude of *V*_oc_ is not only dependent on the contact area and thickness of the gel electrode but also strongly affected by human movement frequency. For example, when the activity changes from walking to running, both the frequency and amplitude of the *V*_oc_ signal change accordingly. Figure 7f,g shows that when the H‐TENG is attached to a joint, it can dynamically capture the bending movements of fingers and elbows and subsequently convert them into voltage signals. The output voltage amplitude increases with the bending angle, because a larger bending angle increases the contact variation between the H‐TENG and the joint surface. Furthermore, when the H‐TENG was subjected to repeated switching cycles between room temperature (25°C) and a deep‑frozen state (−40°C), its hand‐clapping output *V*_oc_ remained relatively stable (Figure S15). This outstanding stability is due to the excellent anti‐freezing property of the hydrogel enabled by the EG‐water binary solvent system, ensuring reliable operation of the H‐TENG for outdoor human motion monitoring in extreme low‐temperature environments.

Taken together, these results showcase the multifunctional prowess of the self‐powered H‐TENG: it not only serves as a sustainable power source to drive electronic components but also enables precise monitoring of distinct joint motion states by capturing the peak features of its electrical output signals. Characterized by exceptional sensing stability, robust operational durability, and superior adaptability to complex environments, the device maintains highly consistent electrical output even under prolonged cyclic operations and diverse external perturbations, validating its profound potential as a reliable self‐powered sensing platform. In essence, as a portable, deformable, and wearable electronic system, the H‐TENG harbors substantial transformative potential in advancing smart sensing technologies and next‐generation mobile power supply solutions.

## 3 Conclusion

In conclusion, this work rationally designed and constructed a multifunctional alkali‐polyphenol (DESL‐OH^−^) synergistic self‐catalytic platform for the rapid room‐temperature free radical polymerization of MAA and HEA monomers in oxygen‐tolerant environments, affording a versatile hydrogel with ultrashort gelation time of merely 2 min. The hydrogel displays remarkable deformability (>900% elongation at break), superior mechanical fatigue resistance (50 consecutive cycles at 400% tensile strain), wide temperature window (−40–25°C), and robust interfacial self‐adhesion performance (0.065 MPa on paper), all of which can be precisely tailored by modulating the DESL content and HEA/MAA mass ratio. Benefiting from the multidimensional catalytic merits of DESL‐OH^−^, alkali‐mediated hydrolysis of HEA is achieved, facilitating the innovative in‐situ generation of EG. This, in turn, confers the hydrogel with exceptional cryo‐tolerance. Endowed with these distinctive attributes, the hydrogel enables integration into an environmentally resilient and highly sensitive TENG (H‐TENG), which delivers stable and long‐term electrical outputs over 10 000 cycles. For H‐TENG, the *V*_oc_, *I*_sc_, and *Q*_sc_ are stably peaked at 200 V, 1.49 µA, and 56.4 nC, respectively, which could be further boosted to 430 V, 2.3 µA, and 133.8 nC under stretching deformation. Notably, the H‐TENG maintains high efficiency of mechanical‐to‐electrical energy conversion even after long‐term cryogenic storage at −40°C, and is capable of directly driving commercial electronic devices, including smartwatches and 18 LEDs in series. This work not only delineates the intrinsic mechanism governing the regulation of gel properties but also puts forward an innovative design paradigm for frost‐tolerant electrode materials, providing critical scientific insights and technical underpinnings to advance the practical applications of TENGs.

## Conflicts of Interest

The authors declare no conflict of interest.

## Supporting information




**Supporting File 1**: advs74924‐sup‐0001‐SuppMat.docx.

**Supporting File 2**: advs74924‐sup‐0002‐VideoS1.mp4.

**Supporting File 3**: advs74924‐sup‐0003‐VideoS2.mp4.

## Data Availability

The data that support the findings of this study are available from the corresponding author upon reasonable request.
