# Supplementary material for: Scalable, Low‐Cost, Freeze‐Resistant Hydrogels by Alkali‐Polyphenol‐Triggered Polymerization for Low‐Temperature Self‐Powered Triboelectric Sensor
Source: Adv Sci (Weinh). 2026 Mar 23;13(31):e74924. doi: 10.1002/advs.74924 (PMC13587990; doi:10.1002/advs.74924)
Supplement: Supplementary file 1 [file ADVS-13-e74924-s003.docx]

**Supporting Information**

**Scalable, Low-Cost, Freeze-Resistant Hydrogels by Alkali-Polyphenol-Triggered Polymerization for Low-Temperature Self-Powered Triboelectric Sensor**

Mengting Zhou^a^, Yaru Bao^a^, Chunpeng Li^a^, Caicai Li^b^, Gaobo Lou^a^, Shaochao Sun^c^, Zhezhe Zhou,^d^ Gaojie Jiao^a,^*, Xiaochun Zhang^a,^*, Pingan Song^d,^*, Dan Sun^a,e,^*

*^a^ College of Chemistry and Materials Engineering, Zhejiang A&F University, Hangzhou 311300, China.*

*^b^ Key Laboratory of Optoelectronic Chemical Materials and Devices-Ministry of Education, College of Optoelectronic Materials and Technology, Jianghan University, Wuhan 430056, China.*

*^c^ Guangxi Key Laboratory of Clean Pulp & Papermaking and Pollution Control, School of Light Industry and Food Engineering, Guangxi University, Nanning 530004, China.*

*^d^ Centre for Future Materials, School of Science, Engineering and Digital Science, University of Southern Queensland, Springfield 4300, Australia.*

*^e^ State Key Laboratory of Advanced Papermaking and Paper-based Materials, South China University of Technology, Guangzhou 510640, China.*

* Corresponding authors.

E-mails: jiaogaojie@zafu.edu.cn (G. Jiao); 20110039@zafu.edu.cn (X. Zhang); pingansong@gmail.com (P. Song); sunday4556@zafu.edu.cn (D. Sun)

**1. Experimental Section**

***Materials***

Sodium hydroxide (NaOH) and ammonium persulfate (APS) were purchased from Sinopharm Chemical Reagent Co., Ltd. α-Methacrylic acid (MAA) was purchased from Shanghai Aladdin Biochemical Technology Co., Ltd. Hydroxyethyl acrylate (HEA) was purchased from Shanghai Macklin Biochemical Co., Ltd.. All the reagents were analytical reagents. Lignin (DESL) was extracted from bamboo using acidic ternary deep eutectic solvents based on a diol protection strategy, as described in our previously published report.^[1]^ The as-prepared DESL possesses a moderate molecular weight (*M*_n_=1700 g mol^-1^, *M*_w_=3740 g mol^-1^) with favorable molecular uniformity (PDI=2.21) and phenolic hydroxyl content (1.52 mmol g^-1^). These structural parameters ensure the high redox activity of DESL, which is crucial for the subsequent self-catalytic polymerization.

***Fabrication of Hydrogel******s***

First, NaOH was dissolved in deionized water, followed by the addition of HEA and MAA. After thorough mixing, DESL was introduced to form a homogeneous mixture designated as solution A. Subsequently, solution B was prepared by dissolving NaOH and APS in deionized water. The two solutions were then combined and vigorously stirred to achieve a uniform mixture, which was poured into PTFE molds and allowed to stand at room temperature for several minutes, yielding the lignin-based hydrogel. The detailed compositions of the hydrogels are provided in **Table S1**.

***Preparation of the H-TENGs***

The H-TENG operating in a single-electrode mode is conceived as a sandwich-type structure, in which the hydrogel serves as the core functional layer, sandwiched between two sheets of triboelectric-active VHB elastomer (3M VHB 9469). Conductive copper wires are fixed to the hydrogel layer to establish electrical connections

***Characterization of the Alkali-polyphenol Synergistic Catalysts***

X-ray photoelectron spectroscopy (XPS) of DESL, NaOH, and APS solid powders was measured using an XPS spectrometer (Thermo Scientific K-Alpha, USA) to identify the oxidation products of DESL. Charge correction was performed using the C-C(H) peak (284.6 eV) in the C 1s spectrum as a reference. 5,5-Dimethyl-1-pyrrolidine-N-oxide (DMPO) spin-trapping electron spin resonance (ESR) spectra were recorded on an ESR spectrometer (Bruker EMXnano) to determine the semiquinone, SO_4_•^-^, and OH• radicals generated by alkali-activated DESL and APS.

***SEM Characterization***

The surface morphology of the freeze-dried hydrogel was characterized using a scanning electron microscope (SEM, JSM-6460LV). Prior to observation, the lyophilized sample was sputter-coated with a thin layer of gold to enhance its electrical conductivity, thereby ensuring high-resolution imaging.

***UV-vis Tests***

Transmittance spectrum of the hydrogels with a thickness of 1 mm in the wavelength range of 200 to 750 nm was obtained using an ultraviolet-visible spectrophotometer (Shimadzu UV-2550).

***Differential Scanning Calorimetry (DSC) Tests***

The anti-freezing property of the hydrogels with different monomer mass ratios was evaluated using a differential scanning calorimetry (DSC 200 F3) instrument under a nitrogen atmosphere. The measurement was performed by first cooling the samples from room temperature to -80 ℃ at a rate of 10 ℃ min^-1^, followed by a 5 min isothermal hold at -80 ℃. The sample was then heated back to room temperature at the same rate. The heat flow during both cooling and heating stages was continuously monitored to evaluate the freezing/melting point of the samples.

***Rheological Analyses***

Each hydrogel was fabricated into circular discs with a thickness of 2 mm and a diameter of 30 mm for rheological characterization. A HAAKE Mars 60 rheometer was used to characterize its rheological behavior. First, frequency sweep test was performed at a fixed strain amplitude of 1%, with the angular frequency ranging from 0.1 rad s^-1^ to 100 rad s^-1^. Subsequently, strain sweep test was conducted at a fixed angular frequency of 10 rad s^-1^, with the strain varying from 0.1% to 100%. Furthermore, to explore the low-temperature rheological properties, temperature sweep test was carried out by cooling the sample from 20 ℃ to -80 ℃ under a fixed angular frequency of 10 rad s^-^¹ and strain of 1%.

***Raman Tests***

Raman spectra of the hydrogels were recorded using a Horiba LabRAM HR Evolution Raman spectrometer (Japan) equipped with a 532 nm laser source.

***Mechanical Measurements***

Mechanical properties of the hydrogels were evaluated using a universal testing machine (CMT-6104) equipped with a 200 N load sensor, with tests conducted at a constant tensile rate of 100 mm min^-1^. All tensile measurements were repeated independently at least three times to ensure data consistency. Uniaxial tensile tests were performed on rectangular specimens with dimensions of 80 mm × 10 mm × 1.5 mm (length × width × thickness). Tensile stress was determined by the ratio of the breaking force to the cross-sectional area. Elastic modulus was derived from the slope of the strain-stress curve within the 0-10% strain range, and toughness was calculated by integrating the area under the stress-strain curve. The calculation of toughness was given as follows. Additionally, for cyclic tensile tests, specimens were stretched to 400% strain and then immediately released, with no interruption between cycles.

$$T=\int_{0}^{\varepsilon} u(\varepsilon)d\varepsilon T Equation.S1$$

In the formula, ε and u(ε) represent the strain and stress (MPa) at the maximum fracture point of the strain-stress curve, respectively.

***Self-Adhesion Evaluation***

To evaluate the adhesive capability of the hydrogels, shear strength tests were conducted on various substrates (wood, rubber, PET, glass, and copper (Cu)) in the laboratory. Prior to each test, a rectangular hydrogel sample was adhered between two substrate layers, with an overlapping adhesion area of 20 mm × 25 mm. The sample was then compressed with a 500 g weight for 12 hours, followed by vertical shear testing at a rate of 100 mm min^-1^. The adhesion strength (T, MPa) was calculated using the following equation.

$$T=\frac{F}{S} Equation. S2$$

In the formula, F and S represent the maximum tensile force (N) and the contact area between the gel and the substrate (cm^2^), respectively.

***Electrical Conductivity***

Electrochemical impedance of the hydrogels was quantified via an electrochemical workstation (CHI660E) using electrochemical impedance spectroscopy (EIS), over the frequency range of 10^6^–10^-1^ Hz at a voltage of 100 mV. Electrical conductivity (σ, mS cm⁻¹) was calculated based on the measured resistance values at different temperatures as follows.

$$\sigma=1000\times\frac{D}{R\times S} Equation. S3$$

In the formula, D, R, and S represent the thickness of the gel (cm), the resistance at room temperature (Ω), and the cross-sectional area (cm^-2^), respectively.

***Finite Element Simulation***

Finite element analysis (FEA) was performed using the Electrostatics (es) module of COMSOL Multiphysics to elucidate the electrical potential distribution between the VHB film and the Al sheet across varying separation distances. A scaled model comprising a VHB dielectric and an Al electrode, each with dimensions of 40 mm in width and 0.5 mm in thickness, was established inside a 70 mm square air domain to minimize edge effects. To simulate triboelectrification, opposing surface charge densities of ± 0.6 × 10^-6^ C m^-2^ were assigned to the VHB and Al surfaces, respectively. By defining the Al electrode as a floating potential, stationary studies and parametric sweeps were executed to determine the potential distribution across varying separation distances and validate the theoretical power generation principles of the H-TENG.

**2. Results and Discussion**

**Figure S1.** Reaction scheme for the in-situ generation of EG via alkali-induced hydrolysis of HEA monomers.

**Figure S2.** (a) The hydrogel gelatinizes rapidly within 2 min at room temperature and (b) can be molded into various shapes.

**Figure S3.** SEM image of the DESL/NaOH@HEA-MAA hydrogel.

**Figure S4.** UV-visible transmittance spectra of the hydrogels with different DESL contents. Insets: Micrograph of a transparent hydrogel (DESL=0.06 wt.%) with a thickness of 1 mm coated onto a mobile phone case.

**Figure S5.** Photograph of the NaOH-free sample after being stored at 25 ℃ for 6 h.

**Figure S6.** Tensile strain and fracture strength of the hydrogels with (a) different DESL contents and (b) different monomer ratios. (c) Segmented non-linear tensile stress-strain curve of the hydrogel with a DESL contents of 0.06 wt.% and a monomer ratio of 3:3, highlighting its mechanical behaviour in two distinct deformation stages of 0-100% and 100-910%. (d) Recovery rates of the hydrogels in continuous cyclic tensile tests.

**Figure S7.** Photographs of the hydrogel adhering to different substrates.

**Figure S8.** The ratio of strong to weak hydrogen bonds in two representative hydrogels calculated based on the fitted peak areas from Raman spectroscopy.

**Figure S9.** (a) Electrochemical impedance spectra and (b) conductivities of the hydrogels with different monomer ratios.

**Figure S10.** (a) Finite element simulation of the potential distribution between VHB film and Al material at different separation distances (5.0 and 10.0 mm). (b, c) Typical output voltage and current curves of H-TENG.

**Figure S11.** *Q*_sc_ responses of the H-TENG assembled from hydrogels with different conductivities.

**Figure S12.** (a) *V*_oc_, (b) *I*_sc_, and (c) *Q*_sc_ responses of H-TENG interfaced with different exogenous materials.

**Figure S13.** *V*_oc_, *I*_sc_, and *Q*_sc_ of the H-TENG (a-c) with different gel areas, (d-f) with different gel thicknesses, and (g-i) under different tensile strains.

**Figure S14.** (a) *V*_oc_, (b) *I*_sc_, and (c) *Q*_sc_ of H-TENG stored at different temperatures.

**Figure S15.** *V*_oc_ of the H‑TENG under hand-clapping motion during repeated freeze-thaw cycles between 25 ℃ and -40 ℃. The gel area is 2 × 3 cm^2^.

.

**Table S1.** The amount of each component added during the preparation of hydrogels.

| **Samples** | **Organohydrogel compositions** | | | | | |
| --- | --- | --- | --- | --- | --- | --- |
|  | **DESL^[a]^ (mg)** | **NaOH**  **(g)** | **MAA^[b]^**  **(g)** | **HEA^[c]^**  **(g)** | **APS^[d]^**  **(mg)** | **Water**  **(g)** |
| 1 | 10 | 2.5 | 3 | 3 | 130 | 8 |
| 2 | 30 | 2.5 | 3 | 3 | 130 | 8 |
| 3 | 60 | 2.5 | 3 | 3 | 130 | 8 |
| 4 | 100 | 2.5 | 3 | 3 | 130 | 8 |
| 5 | 10 | 2.5 | 2 | 4 | 130 | 8 |
| 6 | 10 | 2.5 | 4 | 2 | 130 | 8 |

Note: ^[a]^ Lignin (DESL) was extracted from bamboo using acidic ternary deep eutectic solvents based on a diol protection strategy. ^[b]^*α*-Methacrylic acid. ^[c]^Hydroxyethyl acrylate. ^[d]^Ammonium persulfate.

**Table S2.** Performance comparison of the as-prepared hydrogel and its assembled TENG device with previously reported counterparts.

| **Production cost ($)** | **Self-adhesion (MPa)** | **Frost-resistance (℃)** | **Tensile**  **strain (%)** | **Output**  **voltage (V)** | **Publication** | **Ref.** | **Date** |
| --- | --- | --- | --- | --- | --- | --- | --- |
| **25.6** | **0.065** | **-40** | **918** | **200** | **─** | **This work** | **2026** |
| 44.3 | 0.031 | -40 | 800 | 220 | *Adv. Funct. Mater.* | 2 | 2022 |
| 78.3 | NR | -20 | 349 | 133 | *Adv. Funct. Mater.* | 3 | 2025 |
| 48.6 | 0.054 | -18 | 618 | 19.29 | *Chem. Eng. J.* | 4 | 2025 |
| 104.5 | 0.033 | -10 | 245 | 22.8 | *Int. J. Biol. Macromol.* | 5 | 2025 |
| 66.1 | 0.014 | -26 | 991 | NR | *ACS Nano* | 6 | 2019 |
| 88.0 | NR | -20 | 780 | 0.06 | *ACS Nano* | 7 | 2024 |

NR (not reported).

**Table S3.** Detailed breakdown of raw material costs for the hydrogel.

| **Material** | **DESL** | **NaOH**  **(1000 g)** | **APS**  **(500 g)** | **HEA**  **(100 mL)** | **MAA**  **(100 mL)** | **Deionized water** |
| --- | --- | --- | --- | --- | --- | --- |
| **Added amount (g)** | 0.3 | 75 | 39 | 90 | 90 | 240 |
| **Price ($)** | ─ | 6.3 | 4.9 | 5.8 | 8.6 | ─ |

Note: All other cost estimates are based on current market prices for reagent-grade batches, except for DESL and deionized water.

**References**

[1]	C. Li, M. Zhou, X. Liu, Y. Jiao, Y. Chen, G. Jiao, Q. Sun, F. Zhang, D. Sun, *Ind. Crop. Prod.* **2025**, *230*, 121062.

[2]	D. Sun, Y. Feng, S. Sun, J. Yu, S. Jia, C. Dang, X. Hao, J. Yang, W. Ren, R. Sun, C. Shao, F. Peng, *Adv. Funct. Mater.* **2022**, *32*, 2201335.

[3]	D. Han, Y. Cai, X. Wang, W. Zhang, X. Li, Z. Hou, J. Liu, D. Song, W. Xu, *Adv. Funct. Mater.* **2025**, *35*, 2501362.

[4]	K. Long, Y. Luo, C. Hu, B. Xu, X. Gu, Z. Ding, S. Guo, *Chem. Eng. J.* **2025**, *513*, 162828.

[5]	Y. Tan, Y. Gao, H. Chen, Z. Bi, S. Chen, W. Li, X. Xiao, Z. Ling, *Int. J. Biol. Macromol.* **2025**, *333*, 148912.

[6]	G. Ge, Y. Lu, X. Qu, W. Zhao, Y. Ren, W. Wang, Q. Wang, W. Huang, X. Dong, *ACS Nano* **2019**, *14*, 218.

[7]	Y. Shi, Y. Guan, M. Liu, X. Kang, Y. Tian, W. Deng, P. Yu, C. Ning, L. Zhou, R. Fu, G. Tan, *ACS Nano* **2024**, *18*, 3720.
